# Supplementary figures and images for: Seven-Month Analysis of Five SARS-CoV-2 Antibody Assay Results after ChAdOx1 nCoV-19 Vaccination: Significant Decrease in SARS-CoV-2 Antibody Titer
Source: Diagnostics (Basel). 2021 Dec 30;12(1):85. doi: 10.3390/diagnostics12010085 (PMC8774861; doi:10.3390/diagnostics12010085)

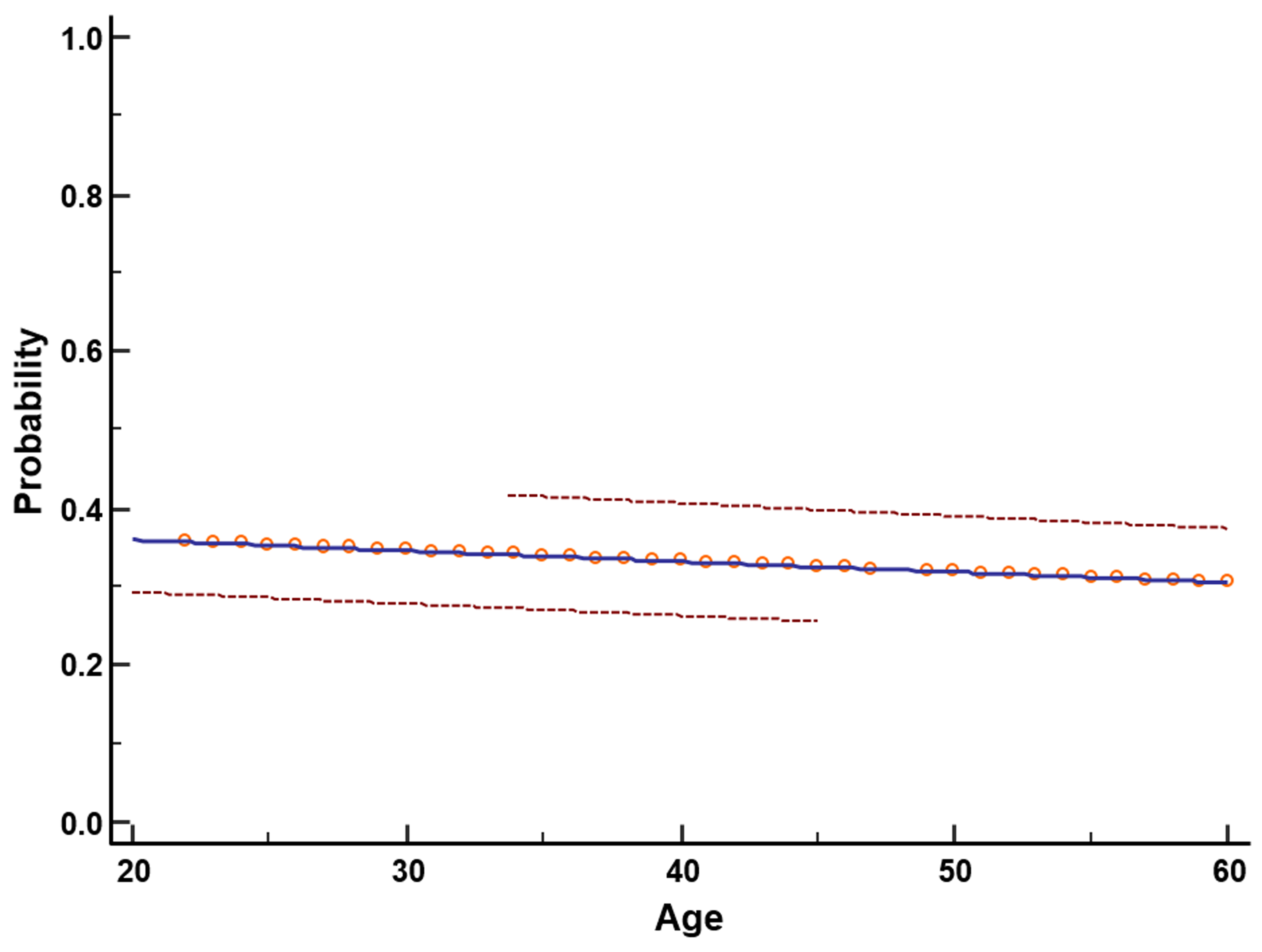

Supplement: Supplementary file 1 [file diagnostics-12-00085-s001.zip › Figure S1.tif]

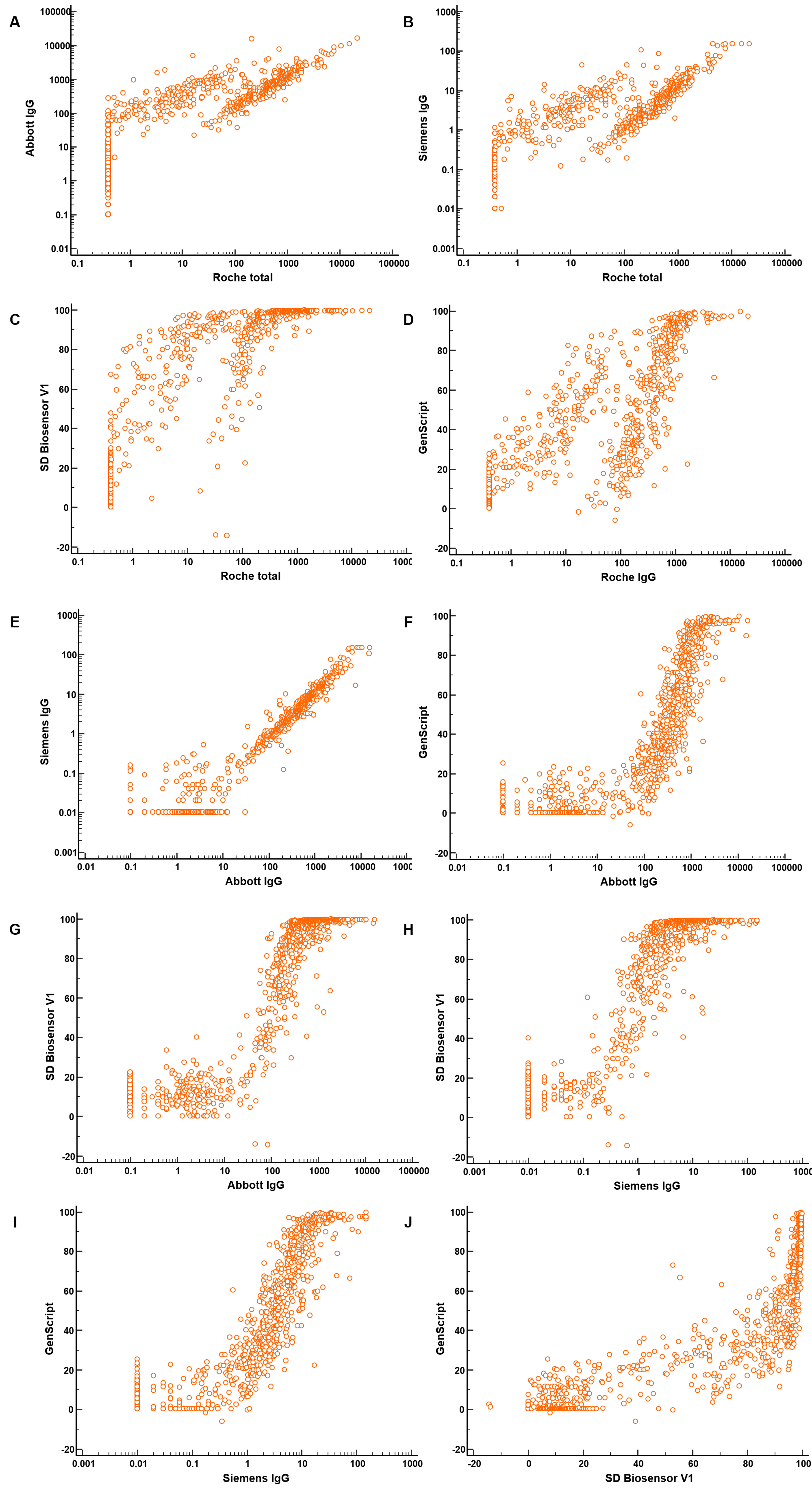

Supplement: Supplementary file 1 [file diagnostics-12-00085-s001.zip › Figure S2.tif]
